# Chronic non-bloody diarrhoea: a prospective study in Malmö, Sweden, with focus on microscopic colitis

**DOI:** 10.1186/1756-0500-7-236

**Published:** 2014-04-14

**Authors:** Johanna K Larsson, Klas Sjöberg, Lina Vigren, Cecilia Benoni, Ervin Toth, Martin Olesen

**Affiliations:** 1 Department of Clinical Sciences, Department of Gastroenterology and Nutrition, Skåne University Hospital, Lund University, Malmö, Sweden; 2 Division of Gastroenterology, Department of Medicine, Trelleborg Hospital, Trelleborg SE-231 85, Sweden; 3 University and Regional Laboratories Region Skåne, Department of Pathology, Skåne University Hospital, Malmö, Sweden

**Keywords:** Collagenous colitis, Diarrhoea, Inflammatory bowel disease, Lymphocytic colitis, Microscopic colitis

## Abstract

**Background:**

Chronic non-bloody diarrhoea affects up to 5% of the population. Microscopic colitis is one of the most common causes, encompassing the subtypes collagenous colitis and lymphocytic colitis. The diagnosis of microscopic colitis is made by histological examination of colonic mucosal biopsy specimens. The aim of this investigation was to determine whether laboratory parameters or questions about disease history or concomitant disease could be helpful in discriminating patients with MC from those with a histologically normal colonic mucosa.

**Findings:**

Patients admitted for colonoscopy because of chronic non-bloody diarrhoea (>2 loose stools for >3 weeks) at endoscopy units in Malmö during 2007 and 2009, were enrolled. A total number of 78 patients were included (60 women, 18 men, median age 59, IQR 45–69 years). Out of these 78, 15 patients (19%) had microscopic colitis (CC; n = 10, LC; n = 5). MC was especially prevalent in patients above the age of 50 (25%). No differences were found between those with normal histology and MC in laboratory analyses (inflammatory and liver parameters). Neither were differences shown in questions regarding symptoms, environmental factors or concomitant diseases except for an association with celiac disease (p = 0.019) and a trend maybe indicating an inverse association with appendectomy (p = 0.057).

**Conclusions:**

Microscopic colitis is associated with female gender, celiac disease and consumption of NSAIDs. Trends were observed indicating that age above 50 years, acute onset and absence of appendectomy may be associated with MC. No associations were observed with other symptoms, calprotectin levels or liver parameters.

## Findings

### Background

Chronic non-bloody diarrhoea (CNBD) is common in Western populations. A prevalence of 5% in the background population and 15% among elderly has been suggested [1]. CNBD is usually defined as more than two loose stools per day for more than four weeks [2]. The underlying disease behind CNBD is not always obvious and can be difficult to diagnose. Besides extra-intestinal causes, several gastrointestinal conditions may be contemplated, such as microscopic colitis (MC), IBS-D (irritable bowel disease with predominantly diarrhoea) or celiac disease (CeD). In patients with other complicating diseases secondary diarrhoea may occur, such as bacterial overgrowth in diabetes or bile acid diarrhoea in Crohn’s disease or as a consequence after cholecystectomy or ileocecal resection [3].

Consequently, even though CNBD may have many causes, MC is important to contemplate since it is diagnosed in 5-20% according to some investigations [4-9]. Histological characteristics of MC are well defined [10]. The condition can be divided into two subgroups; collagenous colitis (CC) and lymphocytic colitis (LC). Collagenous colitis was first described as a case report by CG Lindström in 1976 [11] and LC by Lazenby et al. in 1989 [12]. In MC a macroscopically normal or near normal mucosa is observed [10]. Weight loss and abdominal pain may occur [13,14]. Since it is possible to verify suspected MC first after examination of biopsy specimens any discriminating information in the patient history or laboratory parameters would be helpful.

Occasional previous investigations on patients with active MC have noticed a slight elevation in the calprotectin levels, indicating an inflammatory response [15,16]. Even if this test theoretically could be used for diagnostic purposes it is somewhat complicated and if blood samples could be used instead it would probably be preferred by the majority of the patients. In view of the presence of this low grade inflammation an increase in sedimentation rate, CRP, number of white blood cells or aberrations in the differential count could be contemplated. However, limited data are available regarding laboratory and clinical characteristics in MC. In contrast to classic inflammatory bowel disease (IBD), where disturbed liver function may occur, no information is, to the best of our knowledge, available in MC.

### Research hypothesis

Since MC is manifested through inflammatory activity in colon we imagined that this inflammatory activity could be detected in some way in blood and fecal samples as well. We also imagined that the symptoms, environmental factors, frequency of concomitant diseases (especially diseases considered as autoimmune) as well as characteristics of disease onset would differ from patients with CNBD but with histologically normal colonic mucosa.

### Methods used

Patients admitted for colonoscopy at out-patient clinics (either to private Endoscopy Units in Malmö or to the Endoscopy unit at the Dept of Gastroenterology and Nutrition at Skåne University Hospital, Malmö) because of CNBD from January 2007 until June 2009 were invited to this investigation. CNDB was defined as more than two loose stools per day for more than three weeks, where blood not had been detected. Those willing to participate were asked to answer a questionnaire about their past and present medical history and also left blood and fecal samples.

A self administrated questionnaire about chronic diarrhoea onset (acute or slow), stool frequency (“high” if more than 10 stools/day), stools at night and consistency was completed by the patients. They were also asked questions about recent journeys abroad (i.e. a journey abroad within a month before onset of symptoms) and about “surrounding cases of diarrhoea” (if anyone in the immediate surroundings had had onset of diarrhoea at the same time as the patient). A list of different potential co-factors was also penetrated, such as smoking habits, previous surgery (cholecystectomy or appendectomy), and presence of celiac disease, thyroid disease, rheumatoid arthritis or diabetes mellitus. Medical treatment was also taken into consideration with questions concerning ongoing drug treatment. Three to four years later the patients were asked whether they had recovered or not (total recovery or a reduction of symptoms three to four years after colonoscopy compared to before colonoscopy).

Blood and fecal samples were collected prior to colonoscopy. C-reactive protein (CRP), sedimentation rate, haemoglobin, white blood cells with differential count, platelets, creatinine, aspartate transaminase (AST), alanine transaminase (ALT), alkaline phosphatase (ALP), bilirubin as well as fecal calprotectin were analysed at the Department of Clinical Chemistry, Skåne University Hospital Malmö, according to standardized procedures. Calprotectin was analyzed according to the procedure described by Tøn et al. [17].

During colonoscopy mucosal biopsy specimen were obtained from distal ileum, caecum, ascending colon, right flexure, transverse colon, left flexure, descending colon, sigmoid colon as well as rectum with at least two biopsies from each location.

The histopathological criteria used for CC were:

• A thickened subepithelial collagen layer ≥ 10 micrometers (μm)

• A chronic inflammatory infiltrate in the lamina propria

• Epithelial damage such as flattening and detachment

The histopathological criteria used for LC were:

• Intraepithelial lymphocytes (IEL) ≥ 20 per 100 surface epithelial cells

• A subepithelial collagen layer < 10 μm

• A chronic inflammatory infiltrate in the lamina propria

• Epithelial damage such as flattening and detachment

The specimens were evaluated by a gastrointestinal pathologist with a special interest in MC (M Olesen). The subepithelial collagen layer was measured with an ocular micrometer in a well orientated part of a mucosal section. Measurement of the collagen layer as well as counting of IEL was performed in haematoxylin-eosin. Whenever considered necessary special stainings for collagen fibres (Masson’s trichrome or van Gieson) or reticulin fibres (Sirius red) were carried out. Whenever considered needed sections were immunohistochemically stained with antibodies against T-lymphocytes (CD3) [10]. Diagnoses of ulcerative colitis or Crohn’s disease were set according to established criteria previously described in detail [18].

The investigation was approved by the local Committee of Research Ethics at Lund University (LU: 276/2006). The patients were included first after written informed consent.

For analyses of differences between patients with MC and those with a normal colonoscopy based on macroscopic and microscopic findings (i.e. patients with classic IBD were excluded) Kruskall-Wallis’ non-parametric test was used. Chi2 analysis was used to compare frequencies of MC in different age groups. A p-value below 0.05 was considered significant.

### Results

During the study period 397 patients were admitted for colonoscopy with CNBD. Of these 176 did not answer (mean age 47 years, 108 females (61%)), 110 actively denied participation (mean age 58 years, 80 females (73%)) and 33 cancelled their colonoscopy (mean age 54 years, 20 females (61%)). The mean age of all drop-outs was 52 years, and 65% were females (range 18–89 years). The cohort that finally agreed to participate in the study and completed both colonoscopy and questionnaires consisted of 78 patients, mean age 56 years, 60 women (77%), 18 men (range 18–88 years). The number of included patients that provided blood/fecal samples and answered the questions are stated in Tables 1 and 2.

**Table 1:** Laboratory values in patients with microscopic colitis compared to those with histologically normal colonic mucosa

		**Total number**	**Median**	**IQR**	**Mean**	**Std. Deviation**	**P-values**
**SR**	1	44	9.5	7.25; 17.75	13.2	8.4	
(<20 mm)	2	12	5.5	4.25; 21	10.9	10.3	0.13
**CRP**	1	45	2.5	1.0; 4.9	4.4	5.5	
(<3.0 mg/L)	2	12	1.8	0.72; 4.8	2.6	2.2	0.51
**F-Calprotecin**	1	43	32	20; 66	58.6	66.4	
(<50 μg/g)	2	12	38.5	20; 114	67.8	68.5	0.71
**Haemoglobin**	1	47	137	129; 141	135.3	11.4	
(♀: 117-153 ♂: 134–170 g/L)	2	12	134	127; 142	133.8	10.8	0.87
**Leukocytes**	1	47	6.9	5.7; 7.8	6.9	1.9	
(3.5-8.8 g/L)	2	12	6.8	5.8; 9.5	7.3	2.1	0.74
**Neutrophils**	1	47	4.1	3.2; 5.4	4.4	1.7	
(1.7-8.0 x10^9/L)	2	12	4.0	2.8; 5.7	4.4	1.7	0.98
**Eosinophils**	1	46	0.10	0.10; 0.20	0.2	0.1	
(0.1-0.6 x10^9/L)	2	12	0.10	0.10; 0.20	0.2	0.1	0.56
**Basophils**	1	47	0.10	0.10; 0.10	0.1	0.0	
(<0.2 x10^9/L)	2	12	0.10	0.10; 0.10	0.1	0.0	1.0
**Lymphocytes**	1	47	1.8	1.5; 2.1	1.9	0.6	
(1.1-4.8 x10^9/L)	2	12	2.2	1.9; 2.4	2.2	0.6	0.41
**Monocytes**	1	45	0.50	0.40; 0.60	0.5	0.2	
(0.1-1.0 x10^9/L)	2	12	0.50	0.40; 0.50	0.5	0.2	0.87
**Platelets**	1	47	253	233: 296	273.6	75.3	
(145–348 x10^9/L)	2	11	273	229; 319	271.9	47.9	0.57
**Bilirubin**	1	47	11	9; 13	11.6	3.4	
(5–25 μmol/L)	2	12	12	8; 14	12.3	4.9	0.94
**AST**	1	47	0.39	0.33; 0.45	0.4	0.1	
(0.25-0.75 μkat/L)	2	12	0.42	0.30; 0.46	0.4	0.1	0.87
**ALT**	1	47	0.39	0.33; 0.49	0.4	0.3	
(0.15-1.1 μkat/L)	2	12	0.36	0.22; 0.52	0.4	0.2	0.45
**ALP**	1	44	1.2	1.0; 1.4	1.2	0.3	
(0.60-1.8 μkat/L)	2	12	1.1	0.8; 1.4	1.2	0.4	0.34

1 = normal histology, 2 = MC.

CRP = C-reactive protein, AST = aspartate transaminase ALT = alanine transaminase ALP = alkaline phosphatise.

11 of 58 with normal histology did not leave blood and fecal samples.

3 of the 15 MC-patients did not leave blood and fecal samples.

**Table 2:** Questionnaires’ outcome in patients with microscopic colitis compared to those with histologically normal colonic mucosa

		**Total number**	**Positive answer**	**%**	**P-values**
**Acute onset**	1	56	17	30	
	2	12	7	58	0.068
**High frequency**	1	58	21	36	
	2	13	8	61	0.38
**Watery diarrhoea**	1	58	22	38	
	2	13	7	54	0.51
**Nocturnal diarrhoea**	1	38	5	13	
	2	6	0	0	0.35
**Abdominal pain**	1	58	13	22	
	2	15	1	6.7	0.32
**Surrounding cases**	1	57	1	1.7	
	2	13	0	0	0.63
**Recent journey**	1	58	2	3.4	
	2	12	1	8.3	0.45
**Treatment before**	1	54	12	22	
	2	13	3	23	0.94
**Smoking**	1	54	12	22	
	2	13	4	31	0.18
**Cholecystectomy**	1	49	10	20	
	2	12	3	25	0.73
**Appendectomy**	1	58	18	31	
	2	15	1	6.7	0.057
**Celiac disease**	1	49	1	2.0	
	2	10	2	20	0.019
**Thyroid disease**	1	52	4	7.7	
	2	12	2	17	0.34
**Rheumatoid arthritis**	1	50	2	4.0	
	2	13	1	7.7	0.58
**Diabetes mellitus**	1	52	4	7.7	
	2	12	1	8.3	0.94
**Recovery**	1	39	25	64	
	2	14	10	71	0.90

1 = normal histology, 2 = MC.

The final diagnoses in the cohort are given in Table 3.

**Table 3:** Diagnoses in the patient population

			
Total number	78		
Macroscopic diagnoses	14		
		Crohn´s disease	3
		Ulcerative colitis	2
		Diverticula	5
		Adenoma	4
Microscopic dignoses	16		
		Collagenous colitis	10
		Lymphocytic colitis	5 (1 CeD)
		Celiac disease	2 (1 LC)
Further investigation	16		
		IBS-D	6
		Bile acid diarrhoea	5
		Sjögren’s syndrome	2
		Lactose intolerance	3
Remaining patients	32		
		Recovered	16
		Diagnosis not stated in pat files	16

Among patients below the age of 50, four out of 26 (15%) had classic IBD (two with Crohn’s disease and two with ulcerative colitis) but only two (8%) had MC, one with CC and one with LC. See Table 3 where all diagnoses in the population have been listed. In contrast to this finding, in patients 50 years old or older only one out of 52 had Crohn’s disease (2%) but as many as 13 (25%) had MC; nine CC and four LC (p = 0.067). The age and sex distributions in patients with normal histology and CC and LC, respectively, are depicted in Table 4. As can be seen in this Table the patients with MC were all women compared to 41 out of 58 in those with normal histology (p = 0.017).

**Table 4:** Age in patients with microscopic colitis compared to those with histologically normal colonic mucosa

	**CC**	**LC**	**Normal**
**Total number**	10	5	58
**♀/♂**	10/0	5/0	41/17
**Age at onset (median)**	61	50	55
**IQR**	53, 69	38, 57	34, 65
**Age at colonoscopy (median)**	69	58	59
**IQR**	55, 72	50, 58	46, 69

Patients with MC (n = 15) were compared with patients with normal histology (where also patients with adenoma and diverticula were included, n = 58) regarding laboratory levels and outcome of the questionnaire as stated above. The association with celiac disease was confirmed. There was a trend that patients with MC had an acute disease onset and an inverse correlation with appendectomy. See Tables 1 and 2.

The drugs used by the patients with MC was compared with those in patients with normal histology regarding NSAIDs, ASA, paracetamol, tramadol, antidepressants, PPI, other antacids, medicines regulating blood pressure, diabetes and elevated blood lipids, loperamide, bensodiazepins, folic acid, vitamin B12, calcium and bulking agents. No differences could be observed except for NSAIDs where 9/15 with MC (60%) used NSAIDs compared to 9/56 (16%) in those with normal histology (p = 0.001).

### Discussion

The aim of this study was to find predictors for MC in a population with CNBD. None of the laboratory analyses revealed any differences between patients with MC and those with a histologically normal colonic mucosa (Table 1). Previous investigations have noticed an elevation of calprotectin. Wildt et al. reported a median level of 80 ug/g [16] and Limburg et al. observed a median value of 266 ug/g [15] in active MC. In the present study the median value in active MC was only 38 ug/g compared to 32 in patients with histologically normal colonic mucosa, despite the fact that the same method [17] from Norway has been used in the present report as well as the one by Limburg et al. If this is due to variation because of low sample size or comparison with different cohorts with symptomatic diarrhoea due to other diseases is not possible to conclude from the available data. However, this 36.5-kD nonglycosylated polypeptide trimer [15] is predominantly found in neutrophils, mainly activated in some acute inflammatory responses such as in classic IBD. The fact that we, in our study, could not see any elevation of calprotectin can therefore be explained by the fact that MC is primarily characterized by a chronic inflammation dominated by lymphocytes without any acute inflammatory response including neutrophils. Furthermore, liver disease does not seem to be associated with MC. This observation has not to the best of our knowledge been previously presented elsewhere.

Among patients with MC 31% smoked compared to 22% among those with normal histology although this did not reach statistical significance (p = 0.18). NSAIDs were used to a higher extent among MC patients. This is in accordance with previous studies [19] but also similar to the situation in classic IBD where the risk for active disease is increased if NSAIDs are administered. There was a trend that appendectomy could be inversely correlated with MC (31% in patients with normal histology compared with 6.7% in patients with MC, p = 0.057). This possible association has not been studied extensively but in a population of 130 patients with MC no association was found, in that report neither for cholecystectomy nor for appendectomy [20]. Nevertheless, in view of the association between Crohn’s disease and appendectomy this observation warrants further studies in order to verify if there really is any association or not. Celiac disease was more frequent in MC (p = 0.019), something that has been noticed in other reports as well [13,21].

This investigation has verified a substantial occurrence of MC in a cohort with chronic non-bloody diarrhoea. In 15 patients (19%) collagenous or lymphocytic colitis were revealed (10 with CC and 5 with LC). During the period 1993–97 Fernandez et al. found 24 (6%) with CC and 38 with LC (10%) among 375 patients investigated for chronic diarrhoea [6]. Frequencies of 10% have been suggested in two other investigations; 97 out of 1018 [8] and 27 out of 265 patients [7], respectively. Thijs identified 13 out of 103 (12.6%) [9]. da Silva et al. identified 12% with CC and 7% with LC in 162 patients investigated because of chronic diarrhoea based on the same criteria as ours [4]. Consequently, the proportion of MC in our study is at least in line with the above mentioned previous investigations underlining the fact that MC should be regarded as a possible diagnosis in these patients.

It should be noted that the group investigated in this study consisted of 60 women (77%) with a median age of 60 years, in other words patients where MC is especially prevalent. The demography in our study can be compared to those in some of the other reports; Fernandez investigated a group with a mean age of 56 years, 63% women (16% MC) [6], Thijs a group with a mean age of 45 years, 64% women (13% MC) [9] and da Silva a group with a mean age of 44 years, 59% women (19% MC) [4]. The skewed distribution in age and gender in all these groups reflects the fact that those attending health care because of CNBD primarily are middle-aged or older women.

If the cut-off level is set at 50 years of age the prevalence of MC in patients above this level is markedly high; 25% had MC compared to 8% in those below the age of 50. In this patient group it is thus especially important to consider the possibility of MC. Actually; this particular cut-off level has been suggested previously in a literature review aimed at establishing recommendations for investigation of chronic diarrhoea [22], even though the prevalence increases even more above the age of 70 [8].

A weakness of the present report is the number of included patients. In order to enrol a sufficient number of patients almost 400 patients were invited. A reasonable response rate should have resulted in around 300 patients but for different reasons the final number was below 100. Some cancelled their investigation, some never answered the invitation despite reminders. Yet other actively denied participation and in some cases this could be due to other research projects exhausting the patients. Despite the limited number of participants there are some significant observations that could be of interest for further studies.

### Conclusions

The conclusions of the present report are depicted in Table 5.

**Table 5:** Clinical characteristics associated with MC in patients with CNBD

	
*Significant associations*	Women
	Celiac disease
	NSAID use
*Trends*	Acute onset
	Age above 50 years
	No appendectomy
*No association*	Symptoms except acute onset
	Inflammatory parameters
	Calprotectin
	Liver parameters

## Competing interests

The authors declare that they have no competing interests.

## Authors’ contributions

JKL assisted in data analysis and interpretation and wrote the paper. KS carried out the data collection and analysis, led the writing of the paper. LV assisted in data collection, analysis and interpretation of the results. CB initiated the study, assisted with participant recruitment. ET initiated the study, assisted with participant recruitment. MO initiated the study, assisted with participant recruitment, evaluated the histological specimens, oversaw the study design and assisted with editing the paper. All authors have read, contributed with valuable comments and approved the final version of the paper.
